# Efficacy and tolerability of EPs 7630 in patients (aged 6–18 years old) with acute bronchitis

**DOI:** 10.1111/j.1651-2227.2009.01656.x

**Published:** 2010-04

**Authors:** W Kamin, VG Maydannik, FA Malek, M Kieser

**Affiliations:** 1 University Hospital PaediatricsMainz, Germany; 2 Pediatrics Faculty No. 4, National Medical UniversityKiev, Ukraine; 3 Clinical Research Department, Dr. Willmar Schwabe GmbH & Co. KGKarlsruhe, Germany; 4 Institute of Medical Biometry and Informatics, Ruprecht-Karls-University HeidelbergHeidelberg, Germany

**Keywords:** Acute bronchitis, Bronchitis -specific symptoms, Children and adolescents, EPs-7630, Herbal drug preparation

## Abstract

**Aim:**

For EPs-7630, a herbal drug preparation from *Pelargonium sidoides* roots, therapeutic effects in respiratory tract infections outside the strict indication for antibiotics have already been demonstrated in adults. Now, a dose-finding study for EPs-7630 was performed in children and adolescents.

**Methods:**

A total of 400 patients (aged 6–18 years) were randomized to receive either 30 mg, 60 mg or 90 mg EPs-7630 or placebo daily. Primary outcome criterion was the change in the Bronchitis Severity Score (BSS) from day 0 to day 7.

**Results:**

After 7 days of treatment, the change in the BSS total score was significantly better in the 60 mg and 90 mg groups compared with placebo that of the without relevant differences between these two dosages. Especially ‘coughing’, ‘sputum’ and ‘rales at auscultation’ improved under EPs-7630. Onset of effect was faster, time of bed rest shorter and treatment outcome and satisfaction with treatment were rated better. Tolerability was comparable with placebo in all treatment groups.

**Conclusion:**

EPs-7630 is effective in acute bronchitis outside the strict indication for antibiotics in 6–18 years old patients, with a dose of 60 mg or 90 mg daily offering the best benefit/risk ratio. EPs-7630 significantly reduces the severity of symptoms, leads to a more favourable course of the disease and a faster recovery from acute bronchitis compared with the placebo, and is well tolerated.

## Introduction

Acute bronchitis is one of the most frequent health complaints for which parents seek medical care for their children. According to an extensive evaluation, non-influenza-related viral respiratory tract infections impose a greater economic burden on the public health system than many other clinical conditions (1,2). The common treatment of acute bronchitis with antibiotics has been questioned because of concerns about the emergence of antibiotic resistance and only scant evidence of benefit (3–5). According to expert opinions, antibiotic therapy is mostly ineffective in acute bronchitis, unless the pathogen is of bacterial origin and determined by an appropriate laboratory test (6,7). One therapeutic alternative in the first-line treatment of acute bronchitis outside the strict indication for antibiotics, which has been approved for use in children (≥1 year of age, Germany), is a herbal drug preparation from the roots of *Pelargonium sidoides*, EPs 7630.^1^ Pharmacological activities, including moderate direct antibacterial and antiviral potencies and immunomodulatory capabilities, have been demonstrated *in vitro* for EPs 7630 and its isolated constituents. The immunomodulatory activities are mediated mainly by the release of tumour necrosis factor (TNF-α) and nitric oxides, the stimulation of interferon-β and the increase in natural killer cell activity (8,9). Further biological activities *in vitro* are enhanced phagocytosis, oxidative burst and intracellular killing of human peripheral blood phagocytes (10), improved ciliary beating (11) and inhibition of the interaction between group A streptococci and host epithelia (12). In animal studies, an improvement of sickness behaviour in response to treatment with EPs 7630 has been observed (13). The clinical efficacy and safety of EPs 7630 as a liquid preparation have been demonstrated in adult patients (14–19) as well as in children (19–21) suffering from acute bronchitis or sinusitis. In two observational studies with children suffering from acute bronchitis or severe exacerbation of chronic bronchitis (20,21), EPs 7630 was able to provide complete or partial remission of symptoms in more than 80% of the children, and reduced the severity of symptoms respectively. The tolerability was very good with mild to moderate adverse events (AE) in only 1.8% of patients (21). This study was performed to identify the appropriate dose of EPs 7630 and to demonstrate its efficacy, safety and tolerability in the treatment of patients aged 6–18 years suffering from acute bronchitis.

## Methods

### Study design

The study was performed from February to May 2006 at 16 centres in Ukraine as a randomized, double-blind, placebo-controlled clinical dose-finding study with 4 parallel treatment groups. Individual duration of the study was 7 days. During this time, 3 visits were scheduled (day 0; days 3–5; day 7). The study was conducted according to Good Clinical Practice, the declaration of Helsinki and legal regulations. Approvals of the Ethics Committee and the regulatory authorities were obtained prior to the start of the clinical trial. To ensure the integrity and validity of the study, an independent Case Assessment and Data Quality Evaluation Committee (CADQEC) supervised the quality of the data generated at the trial sites before and after unblinding.

### Patients

Male or female patients aged 6–18 years old suffering from acute bronchitis with symptoms starting ≤48 h prior to inclusion in the study and with a total score of bronchitis-specific symptoms (BSS) ≥5 points at screening were included in the study. Major exclusion criteria were: treatment with antibiotics, bronchodilators or glucocorticoids during the last 4 weeks, or with analgetics, secretolytics, mycolytics or antitussiva during the last 7 days prior to study inclusion; indication for treatment with antibiotics; allergic asthma; tendency to bleed; severe heart, renal or liver diseases and/or immunosuppression, known hypersensitivity against *P. sidoides*; chronic obstructive pulmonary disease and pregnancy. Patients and their legal representatives respectively gave their written informed consent in accordance with the legal requirements.

### Study medication

After written informed consent, eligible patients were randomly allocated to one of four treatment groups in a balanced way (with a block size of four), according to a computer-generated randomization list. This randomization list was prepared by using the validated random number generator R-Code. The length of the balanced blocks was fixed in a separate document that was withheld from the study sites. The investigators received sealed emergency envelopes for individual patients, all of which were returned unopened after completion of the trial.

Patients were given EPs 7630, a herbal drug preparation from the roots of *P. sidoides* (1:8–10), dried, extraction solvent: ethanol 11% (w/w), as EPs 7630 film-coated tablets [3 × 10 mg (= 30 mg group), 3 × 20 mg (= 60 mg group) or 3 × 30 mg/day (= 90 mg group) EPs 7630] 30 min before or after a meal for 7 consecutive days, or a matched placebo for the same time period.

### Measurements

The *primary efficacy variable* was the change in the BSS total score from day 0 to day 7 rated by the investigator. The BSS total score consists of the five symptoms coughing, sputum production, pulmonary rales at auscultation, chest pain while coughing and dyspnoea, which are the most important features associated with acute bronchitis (22,23), rated on a scale from 0 (not present) to 4 (very severe) and leading to a maximum total score of 20 points.

*Secondary efficacy variables* were: treatment response according to three criteria (BSS total score of <3 on day 7, decrease in BSS total score of at least 7 points from day 0 to day 7 and BSS total score <3 on day 7 combined with a decrease in BSS total score of at least 7 points from day 0 to day 7), onset of effect, change in the total score of individual symptoms and, change of general symptoms (e.g. ‘absence of appetite’, ‘headache’ and ‘vomiting’) and health status of patients using the questionnaires for health state of children (FGK, *Fragebogen zum Gesundheitszustand für Kinder*). Additional parameters were bed rest duration and ability to attend kindergarten, school or work. *Treatment outcome* was assessed by both the investigator and the patient using the Integrative Medicine Outcomes Scale (IMOS) consisting of a 5-point rating scale (1 = ‘complete recovery’, 2 = ‘major improvement’, 3 = ‘slight to moderate improvement’, 4 = ‘no change’ and 5 = ‘deterioration’). *Satisfaction with treatment* was assessed using the Integrative Medicine Patient Satisfaction Scale (IMPSS), a five-point scale comprising the ratings: 1 = ‘very satisfied’, 2 = ‘satisfied’, 3 = ‘undecided’, 4 = ‘dissatisfied’ and 5 = ‘very dissatisfied’. Safety parameters were surveillance of AEs, laboratory safety parameters and vital parameters. Prior to unblinding, every AE was classified by the investigator in one of four categories according to the data available with regard to the possible causal relationship to the administration of the study medication (probable – possible – unlikely – no relationship).

### Statistical methods

The primary objective of this study was to demonstrate the efficacy and to evaluate the safety and tolerability of three doses of EPs 7630 in acute bronchitis. As a result of the lack of empirical data, the form of the dose–response relationship was unknown. As a consequence, there was high uncertainty about the assumptions to be made for sample size calculation and whether the desired power would actually be achieved with a fixed sample size design. Thus, the study was planned and performed with an adaptive interim analysis (24). The primary outcome variable for confirmatory treatment group comparisons of efficacy was the intra-individual difference of the BSS total score between day 0 and day 7. A closed testing procedure was applied as follows (25). In the first step, differences between placebo and the three active dose levels were investigated. If the corresponding null hypothesis (no difference between the active dose levels and placebo) could be rejected, the null hypotheses stating no difference between placebo and two active dose levels were tested for all subsets of two active dose levels. For active dose levels for which all related null hypotheses could be rejected, the null hypotheses stating no difference between the active dose level and placebo were tested. The global null hypotheses (placebo vs. 30 mg vs. 60 mg vs. 90 mg and placebo vs. 30 mg vs. 60 mg; placebo vs. 30 mg vs. 90 mg; placebo vs. 60 mg vs. 90 mg) were tested using the Bartholomew test for unknown but common variances (26). The three single null hypotheses comparing each of the active dose levels with placebo were tested with an analysis of covariance (ANCOVA), with the factors ‘treatment group’ and ‘centre’ and the covariate ‘baseline value of the total score of BSS’. The one-sided boundaries for early rejection or acceptance of null hypotheses within the interim analysis were α_1_ = 0.0152 and α_0_ = 0.20 respectively (overall one-sided type I error rate α = 0.025) (24).

Regarding the secondary efficacy variables, descriptive statistical methods were used for the comparison of treatment groups and accordingly, the resulting p-values have to be interpreted in an exploratory manner. All statistics are based on the full analysis set according to the intention-to-treat principle using the last observation carried forward method for missing values.

## Results

### Disposition of patients and baseline characteristics

A total of 400 patients were included for screening and were subsequently randomized to receive 30, 60 or 90 mg EPs 7630 or matching placebo daily. All patients were included in the safety analysis. One patient in the 30 mg group could not be analysed for efficacy because of early dropout without any post-baseline measurement (withdrawal of consent). Thus, the full analysis set comprised 399 patients; 101 patients received placebo, 100 patients received 30 mg, 99 patients received 60 mg and 99 patients received 90 mg EPs 7630. The evaluation of baseline data (Table 1) revealed no noticeable differences between the treatment groups at baseline. Almost all patients took the medication exactly as prescribed. The mean treatment duration was about 7 days in all groups.

**Table 1:** Baseline data (mean ± SD or relative frequencies)

	Placebo n = 101	EPs 7630 (30 mg/day) n = 100	EPs 7630 (60 mg/day) n = 99	EPs 7630 (90 mg/day) n = 99
Gender				
Male	50.5%	53.0%	51.5%	52.5%
Female	49.5%	47.0%	48.5%	47.5%
Age (years)	12.7 ± 3.7	12.5 ± 3.5	12.9 ± 3.7	12.6 ± 3.7
Weight (kg)	47.6 ± 15.1	46.4 ± 13.5	47.1 ± 13.7	47.0 ± 15.3
Height (cm)	155.6 ± 18.5	154.4 ± 17.4	155.7 ± 16.4	154.4 ± 17.3
BMI (kg/m^2^)	19.0 ± 2.8	18.9 ± 2.4	19.9 ± 2.6	19.1 ± 3.5
BSS total score: individual symptoms	2.5 ± 0.5	2.5 ± 0.6	2.4 ± 0.6	2.6 ± 0.6
Coughing	0.8 ± 0.7	0.8 ± 0.7	0.8 ± 0.7	0.8 ± 0.8
Sputum production	2.0 ± 0.5	2.0 ± 0.6	1.9 ± 0.5	2.0 ± 0.6
Pulmonary rales at auscultation	1.4 ± 0.7	1.4 ± 0.7	1.3 ± 0.7	1.3 ± 0.7
Chest pain while coughing Dyspnoea	0.2 ± 0.5	0.2 ± 0.5	0.3 ± 0.5	0.3 ± 0.5
BSS total score	6.8 ± 1.4	6.9 ± 1.6	6.8 ± 1.5	7.0 ± 1.5
Patients with BSS total score ≥7	53.5%	54.0%	55.6%	59.6%

### Primary outcome measure

The decrease in the *BSS total score* between day 0 and day 7 was more pronounced in the active treatment groups compared with that in the placebo group [placebo: 3.3 ± 2.6, EPs 7630 (30 mg): 3.6 ± 2.4, EPs 7630 (60 mg): 4.4 ± 2.4, EPs 7630 (90 mg): 5.0 ± 1.9]. The confirmatory aim of the study was already reached at the interim analysis: All global null hypotheses comparing placebo with all three or to combinations of two active dose levels could be rejected (each p < 0.0001 except for the comparison placebo vs. 30 mg vs. 60 mg EPs 7630 with p = 0.0011, one-sided Bartholomew tests). The subsequent pairwise comparisons of each active treatment group with placebo using the ANCOVA model revealed statistically significant differences in the decrease in the BSS total score for the EPs 7630 60 mg and 90 mg groups (p = 0.0004 and p < 0.0001 respectively, two-sided ANCOVA p-values).

**Figure 1:**
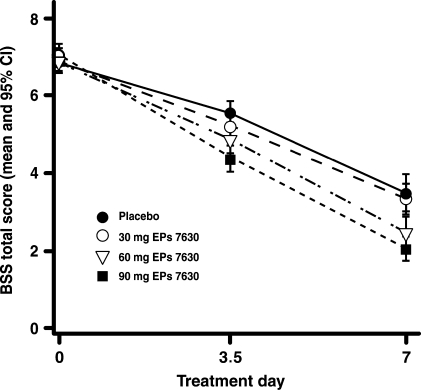
Course of the total score of bronchitis-specific symptoms from day 0 to day 7.

A considerable difference in the BSS total score for the EPs treatment groups was already observed on days 3–5 and increased – in a dose-dependent manner – further until day 7, especially for the dosages of 60 mg and 90 mg. The courses of BSS total scores together with the respective 95% confidence intervals are shown in Figure 1.

### Secondary outcome measures

**Figure 2:**
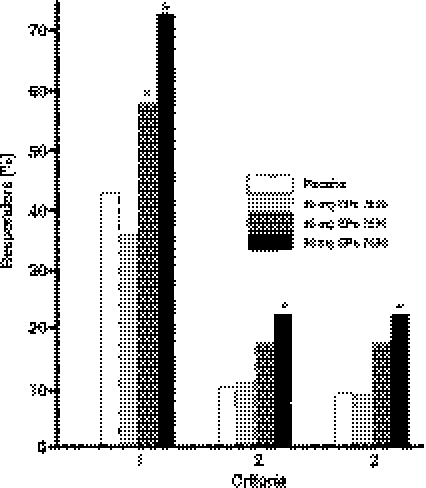
Treatment response. Frequency of responders for 3 criteria: criterion 1: BSS total score <3 points at day 7 (*p = 0.0339 for 60 mg EPs 7630 and p = 0.0001 for 90 mg EPs 7630 compared with placebo); criterion 2: decrease in BSS total score of at least 7 points from day 0 to day 7 (*p = 0.0175 for 90 mg EPs 7630 compared with placebo); criterion 3: combination of criteria 1 and 2 (*p = 0.0093 for 90 mg EPs 7630 as compared with placebo) (two-sided χ^2^-test, each).

*Treatment response* calculated on the basis of the BSS total scores was higher in the active treatment groups than in the placebo group (Fig. 2). Statistically significant differences regarding criterion 1 were determined for the 60 mg and 90 mg EPs 7630 groups in comparison with placebo. Regarding criteria 2 and 3, a significant difference in the rate of responders compared with placebo was observed for the 90 mg EPs 7630 group.

**Figure 3:**
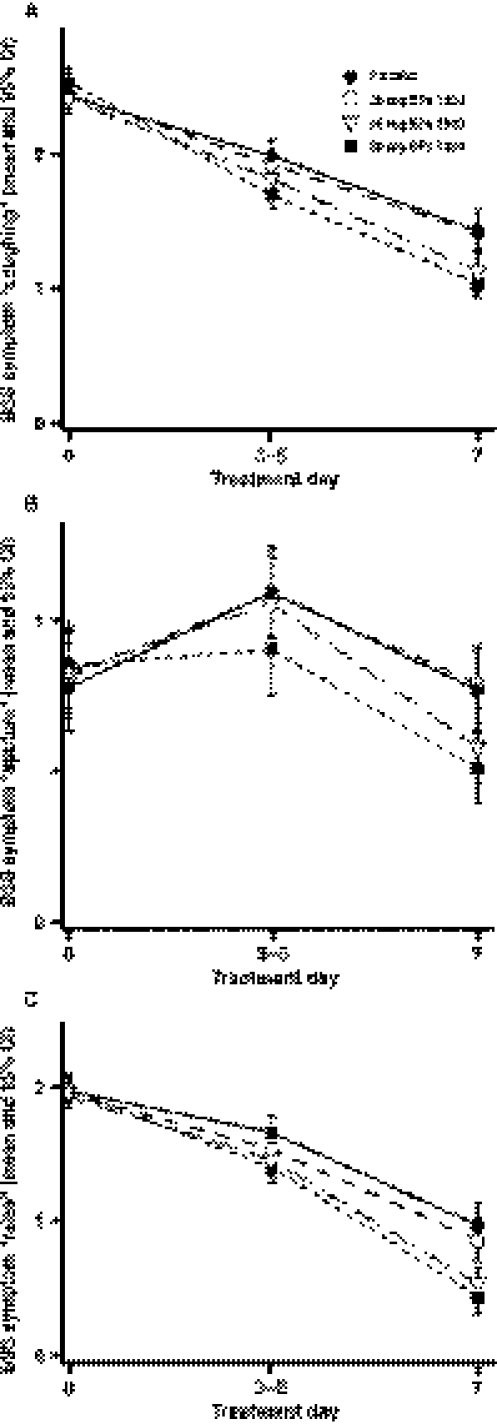
Courses of the bronchitis-specific symptoms ‘coughing’ (a), ‘sputum’ (b) and ‘pulmonary rales at auscultation’ (c) from day 0 to day 7.

The mean decrease in the *individual BSS*‘coughing’, ‘sputum’, ‘pulmonary rales at auscultation’, ‘chest pain while coughing’ and ‘dyspnoea’ from day 0 to day 7 was markedly more pronounced in the EPs 7630 (60 mg) and EPs 7630 (90 mg) groups than in the placebo group. The active treatment groups showed a significant dose-dependent advantage compared with placebo for the symptoms ‘coughing’ (p < 0.0001), ‘sputum’ (p = 0.0016) and ‘pulmonary rales at auscultation’ (p < 0.0001) (Fig. 3). Pairwise comparisons with placebo showed statistically significant advantages of EPs 7630 in the 60 mg and 90 mg group for the symptoms ‘coughing’ (p = 0.0433 and p = 0.0002 respectively), ‘sputum’ (p = 0.0499 and p = 0.0048 respectively) and ‘pulmonary rales at auscultation’ (p = 0.0014 and p < 0.0001 respectively, two-sided *t*-test, each).

A statistically significant dose-dependent effect of EPs 7630 on the *general symptoms*‘absence of appetite’ (p = 0.0234), ‘headache’ (p = 0.0112), ‘vomiting’ (p = 0.0142) from day 0 to day 7 could also be found (Bartholomew test). This was confirmed by pairwise comparisons with placebo, which revealed a significant advantage in the EPs 7630 (90 mg) group regarding the general symptoms ‘absence of appetite’ (p = 0.0128) and ‘headache’ (p = 0.0090).

**Figure 4:**
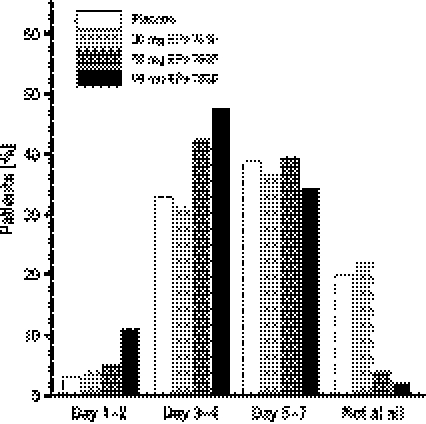
Day of onset of treatment effect as reported by the patient.

The rate of patients in the EPs 7630 (60 mg) and EPs 7630 (90 mg) groups reporting the *onset of effect* before day 5 was higher than that in the placebo group. A statistically significant advantage regarding the onset of effect in the EPs 7630 (60 mg) and EPs 7630 (90 mg) groups could be demonstrated (p = 0.0060 and p < 0.0001 respectively, two-sided Mantel–Haenszel χ^2^-test) (Fig. 4).

**Figure 5:**
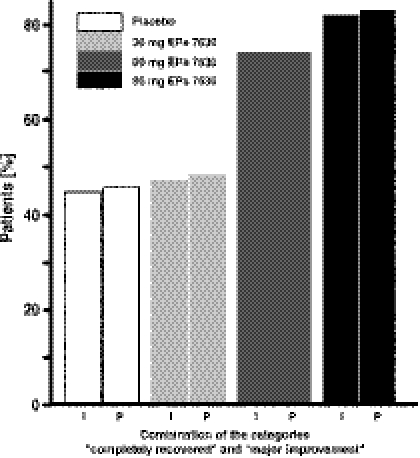
Improvement of the treatment outcome. Percentage of patients judged as completely recovered or showing major improvement by the investigator (I) and patient (P).

*Treatment outcome* as evaluated by the investigators and patients by means of the IMOS showed a significantly better outcome compared with placebo (Fig. 5). The *satisfaction of patients* according to the IMPSS was also better in the EPs 7630 groups. Patients in the active treatment groups (57.0% for EPs 7630 30 mg, 66.7% for EPs 7630 60 mg and 81.8% for EPs 7630 90 mg) were more often satisfied or very satisfied than those in the placebo group (45.5%).

In all groups, an improvement in *health status* as assessed using the FGK questionnaire was observed between day 0 and day 7. This improvement was more pronounced in the EPs 7630 (60 mg) and EPs 7630 (90 mg) groups compared with that in the placebo. A pairwise comparison with placebo (two-sided *t*-test) revealed a statistically significant advantage of the EPs 7630 (60 mg) and EPs 7630 (90 mg) group over placebo for the FGK item ‘I am feeling ill’ (p = 0.0012 and p = 0.0001 respectively). In addition, the EPs 7630 (90 mg) group showed a significant improvement over placebo for the items ‘I have trouble playing or learning’ and ‘I sleep bad’ (p = 0.0063 and p = 0.0067 respectively).

Between day 0 and day 7, the number of *patients able to attend kindergarten, school or work* improved markedly in all groups, especially in the EPs 7630 (60 mg) and EPs 7630 (90 mg) groups. At day 0, only 1 patient (1%) was able to attend kindergarten, school or work in the placebo and 60 mg group respectively. At day 7, 33.7% (placebo), 35.0% [EPs 7630 (30 mg)], 44.4% [EPs 7630 (60 mg)] and 53.5% [EPs 7630 (90 mg)] of patients had regained this ability.

### Safety analysis

A total of 80 AEs were observed in 77 of 400 patients (19.3%). The most frequent AEs were gastrointestinal disorders (11%), with 22.8% [23 AEs in 23 patients; EPs 7630 (30 mg) group], 17.2% [20 AEs in 17 patients; EPs 7630 (60 mg) group] and 19.2% [19 AEs in 19 patients; EPs 7630 (90 mg) group] respectively. The frequency of AEs in the active treatment groups was 17.8% (18 AEs in 18 patients), which was similar to that in the placebo. None of the AEs was classified as serious. The frequency of patients with suspected drug-related AEs was 5.9% [6 of 101 patients; EPs 7630 (30 mg) group], 5.1% [5 of 99 patients; EPs 7630 (60 mg) group], 5.1% [5 of 99 patients; EPs 7630 (90 mg) group] and 4.8% (5 of 101 patients; placebo group). With 0.008, 0.008 and 0.007 events/days of exposure, the incidence of AEs in the active treatment groups was in the range of that of placebo (0.006 events/days of exposure), including their putative causal relationship to the study medication.

## Discussion

The results of this study demonstrate the efficacy, tolerability and safety of EPs 7630, a herbal drug preparation from *P. sidoides* roots, administered as film-coated tablets in 6–18 years old patients. The primary outcome parameter, i.e. improvement in the BSS total score from day 0 to day 7, was superior in the EPs 7630 (60 mg) and the EPs 7630 (90 mg) groups than that in the placebo group.

As the definition of acute bronchitis is still under discussion and diagnosis is solely based on clinical findings, without standardized diagnostic signs and sensitive or specific confirmatory laboratory tests (3,27), the development of commonly accepted diagnostic criteria and validated scores is desirable. As a result of the current lack of standardized criteria, all outcomes applied in this trial are subjective. BSS, FGK, IMPSS and IMOS used in this study are not validated but appear to be associated with a clinical benefit, as already shown in former trials (14,15).

The treatment groups were well balanced with respect to baseline data. Family smoking was not documented in this study, but patients with diseases known to be caused or exacerbated by environmental tobacco smoke (e.g. allergic asthma, COPD) were excluded from study participation.

The improvement in the individual symptoms as well as other secondary outcome measures was clearly more pronounced in the active treatment groups of 60 mg and 90 mg EPs 7630 per day. Regarding the individual BSS for instance, the benefit was most pronounced for the symptoms ‘coughing’ and ‘pulmonary rales at auscultation’, which can be explained by an improvement in ciliary beating found in an *in vitro* study (11). This could be an important mode of action independent of antibacterial activity, because most episodes of acute bronchitis are of viral origin (3,28). A benefit in bacterial infections may be achieved by the inhibitory effect of EPs 7630 on the interaction between bacteria and epithelia (12,29). Therefore, EPs 7630 could be considered as first-line treatment for all types of bronchitis, because even in diseases needing antimicrobial therapy, this initial treatment can bridge the time between presentation of the patient and the final decision on an appropriate antibiosis, thus reducing the risk of uncritical antibiotic treatment. Patients would benefit from fast improvement of symptoms irrespective of the actual cause of bronchitis, be it of bacterial or viral origin.

The generally low incidence and severity of AEs in all dose groups of EPs 7630 indicate a good tolerability and safety also with higher concentrations of EPs 7630 in the study population of this clinical trial.

This study confirms former results on the efficacy of EPs 7630 (14–21). In contrast to the previous studies performed in children, which were conducted as open-label, post-marketing surveillance studies (18–21), this randomized, double-blind, placebo-controlled study describes the first clinical dose finding with EPs 7630 in schoolchildren and adolescents with acute bronchitis. Based on the efficacy and safety results presented, a daily dose of 60 mg EPs 7630 could represent the optimal dose with respect to the benefit/risk ratio.

## Footnotes

1 EPs® 7630 is the active ingredient of the product Umckaloabo® (ISO Arzneimittel, Ettlingen, Germany).

## Abbreviations

AE: adverse event
ANCOVA: analysis of covariance
BSS: bronchitis-specific symptoms
EPs 7630: herbal preparation from the roots of *Pelargonium sidoides* (1:8–10, extraction solvent: ethanol 11% (w/w): registered trademark in Germany)
FGK: Fragebogen zum Gesundheitszustand für Kinder (questionnaire for health state of children)
IMOS: integrative medicine outcomes scale
IMPSS: integrative medicine patient satisfaction scale
